# Dynamics of Cardiometabolic Risk Factors Are Linked to the Risk of Hypertension and Diabetes in MASLD

**DOI:** 10.1002/kjm2.70077

**Published:** 2025-07-12

**Authors:** Chin‐I Shih, Ming‐Lun Yeh, Yi‐Hung Lin, Ping‐Tsung Shih, Kuan‐Ta Wu, Meng‐Hsuan Hsieh, Jeng‐Fu Yang, Yi‐Yu Chen, Po‐Cheng Liang, Yu‐Ju Wei, Pei‐Chien Tsai, Ya‐Yun Cheng, Ming‐Yen Hsieh, Chih‐Wen Wang, Chung‐Feng Huang, Jee‐Fu Huang, Chia‐Yen Dai, Chi‐Kung Ho, Wan‐Long Chuang, Vincent Wai‐Sun Wong, Wei‐Ting Chang, Ming‐Lung Yu

**Affiliations:** ^1^ Tri‐Service General Hospital, National Defense Medical Center Taipei Taiwan; ^2^ School of Medicine and Doctoral Program of Clinical and Experimental Medicine College of Medicine and Center of Excellence for Metabolic Associated Fatty Liver Disease, National Sun Yat‐Sen University Kaohsiung Taiwan; ^3^ Hepatobiliary Division, Department of Internal Medicine and Hepatitis Center Kaohsiung Medical University Hospital, Kaohsiung Medical University Kaohsiung Taiwan; ^4^ Center of Hepatitis Research College of Medicine and Center of Metabolic Disorders and Obesity, Kaohsiung Medical University Kaohsiung Taiwan; ^5^ Department of Internal Medicine Kaohsiung Medical University Hospital, Kaohsiung Medical University Kaohsiung Taiwan; ^6^ Department of Preventive Medicine, and Health Management Center Kaohsiung Medical University Hospital, Kaohsiung Medical University Kaohsiung Taiwan; ^7^ Mok Hing Yiu Professor of Medicine, Department of Medicine and Therapeutics Faculty of Medicine, Chinese University of Hong Kong Hong Kong China; ^8^ Division of Cardiology, Department of Internal Medicine Chi‐Mei Medical Center Tainan City Taiwan

**Keywords:** cardiometabolic risk factors, diabetes, hypertension, MASLD, metabolic dysfunction‐associated steatotic liver disease

## Abstract

This study investigates the impact of cardiometabolic risk factors (CMRF) on the prevalence and incidence of hypertension (HTN) and diabetes mellitus (DM) in individuals with metabolic dysfunction‐associated steatotic liver disease (MASLD) and nonsteatotic liver disease (non‐SLD), using both cross‐sectional and longitudinal data. A total of 32,569 Taiwanese adults without viral hepatitis or significant alcohol consumption who underwent health checkups from 1999 to 2013 were analyzed cross‐sectionally. Among them, 27,109 individuals free of HTN and DM at baseline and within 1 year of enrollment were followed longitudinally. Participants were classified into four groups based on hepatic steatosis assessed by ultrasound and presence of CMRF: healthy control (non‐SLD/CMRF‐), simple SLD (SLD/CMRF‐), non‐SLD/CMRF+, and MASLD. MASLD patients exhibited markedly higher annual incidence rates of HTN and DM (19.7 and 6.3 per 1000 person‐years) compared to non‐SLD individuals (HTN: 9.0; DM: 0.6 per 1000 person‐years). The risk of incident HTN and DM increased progressively with the number of CMRF, with adjusted hazard ratios (aHR) ranging from 2.02 to 15.53 for HTN and from 2.92 to 82.38 for DM. Regression of cardiometabolic dysfunction decreased the risk of HTN and/or DM, and vice versa. The presence of CMRF significantly increased the likelihood of developing HTN and DM in both SLD and non‐SLD groups, with aHRs up to 7.48 for HTN and 15.38 for DM. In conclusion, MASLD is strongly associated with increased prevalence and incidence of HTN and DM, and the burden and trajectory of CMRF critically modulate these risks.

**Abbreviations**
aHR: adjusted hazard ratio
ALT: alanine aminotransferase
anti‐HCV: HCV antibody
aOR: adjusted odds ratio
AST: aspartate aminotransferase
BMI: body mass index
cHR: crude hazard ratio
CIs: confidence intervals
CMD: cardiometabolic dysfunction
CMRF: cardiometabolic risk factors
DBP: diastolic blood pressure
DM: diabetes
FG: fasting glucose
FL: fatty liver
FLD: fatty liver disease
HbA1c: hemoglobin A1C
HBsAg: HBV surface antigen
HDL‐C: high‐density lipoprotein cholesterol
HTN: hypertension
LDL‐C: low‐density lipoprotein cholesterol
MASLD: metabolic dysfunction‐associated steatotic liver disease
NAFLD: nonalcoholic fatty liver disease
ORs: odds ratios
SBP: systolic blood pressure
SLD: steatotic liver disease
T2DM: Type 2 DM
USG: ultrasound sonography

## 1 Introduction

Nonalcoholic fatty liver disease (NAFLD) is considered the hepatic component of a multisystem disease stemming from insulin resistance and is highly associated with metabolic syndrome, including hypertension (HTN), Type 2 diabetes mellitus (DM), obesity, and dyslipidemia [1, 2, 3]. In our previous research targeting the general population in Taiwan, a strong relationship between the severity of fatty liver (FL) and the risk of HTN and DM was observed, with a 1.26‐ and 1.54‐fold risk of new‐onset HTN and a 3.22‐ and 5.88‐fold risk of new‐onset DM for mild FL and moderate/severe FL patients, respectively [4]. Furthermore, we found that the regression or resolution of FL during follow‐up reduced the risk of HTN and/or DM. This highlights the critical importance of early intervention for FL through lifestyle adjustments, as well as the essential need for regular monitoring of blood pressure and glucose levels for individuals with FL.

Recently, the term “steatotic liver disease” (SLD) was introduced as a comprehensive term encompassing all causes of liver steatosis [5]. Furthermore, a new nomenclature of SLD known as metabolic dysfunction‐associated steatotic liver disease (MASLD) has emerged, replacing the term NAFLD [5]. This designation has been established not by exclusion of other liver diseases, but rather by the presence of hepatic steatosis alongside specific, primarily cardiometabolic risk factors (CMRFs) [6, 7]. This evolution in terminology, encompassing MASLD, may help identify individuals with potential metabolic dysfunction as the main cause of hepatic steatosis. This characterization not only enhances disease recognition, mitigates social stigma, but also expedites advancements in drug development and biomarker identification. However, the impact of this new nomenclature on management of what was previously referred to as NAFLD among diverse countries and ethnicities worldwide still lacks comprehensive information. Furthermore, impact of cardiometabolic components among patients with SLD in the risk of HTN and DM has not been evaluated.

In the current study, we aimed to investigate the impact of cardiometabolic burdens and dynamic in the risk of HTN and DM among patients with MASLD, compared to the counterparts. To this aim, we adopted a retrospective cross‐sectional and longitudinal cohort designed to investigate the risk factors associated with co‐existing (prevalent) and new‐onset (incident) HTN and DM among patients with MASLD.

## 2 Materials and Methods

### 2.1 Population

A total of 225,308 subjects who visited the Department of Preventive Medicine and Health Management Center, Kaohsiung Medical University Hospital (KMUH) for health checkups between 1999 and 2013 were screened for the current study. Information identifying the subjects was eliminated from the dataset before commencement. The study was approved by the institutional review board of KMUH.

### 2.2 Cross‐Sectional Cohort

**FIGURE 1:**
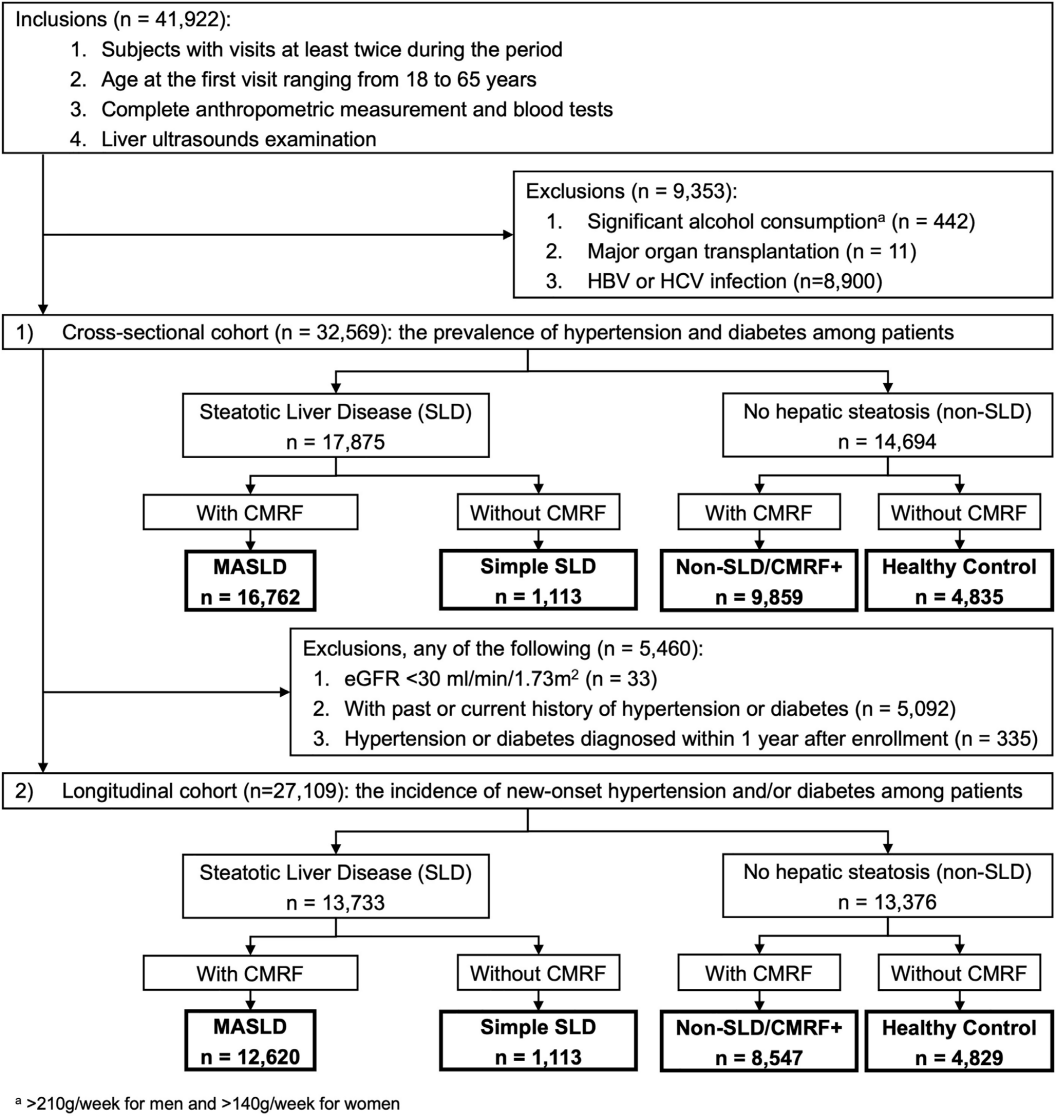
Flow chart for the study cohorts and the definition of SLD subtypes. A total of 41,922 subjects (259,927 person‐visits) met the inclusion criteria. There were 32,569 subjects (195,839 person‐visits) in the cross‐sectional cohort and 27,109 subjects (159,034 person‐visits) in the longitudinal cohort.

A total of 41,922 subjects (259,927 person‐visits), who visited the department at least twice, aged 18–65 years during the first visit, and had complete data of anthropometric measurement, blood tests, and liver ultrasound sonography (USG), were enrolled in the study. Subjects with significant alcohol consumption (*n* = 442, estimated based on self‐reported data on the amount and frequency of alcohol use within the previous year; consider significant if > 210 g/week for men and > 140 g/week for women) [8], with a history of major organ transplantation (*n* = 11), and with HBV and/or HCV infection (*n* = 8900) were excluded. Consequently, the cross‐sectional cohort consisted of 32,569 subjects to evaluate the correlation between cardiometabolic components in MASLD and the prevalence of existing HTN and DM at enrollment (Figure 1).

### 2.3 Longitudinal Cohort

To evaluate incidence of new‐onset HTN and/or DM among patients, 5092 subjects with HTN and/or DM at baseline and 335 subjects with HTN and/or DM diagnosed within 1 year after enrollment, and 33 subjects with an estimated glomerular filtration rate < 30 mL/min/1.73 m^2^ were excluded. Finally, 27,109 subjects were included as the longitudinal cohort to evaluate the incidence of new‐onset HTN and DM (Figure 1).

### 2.4 Assessment of Hepatic Steatosis

Abdominal USG was performed at KMUH, using Toshiba SSA‐250, SSA‐520A, and SSA‐660A devices with 3.5 MHz convex transducers (Toshiba, Tokyo, Japan). The presence of hepatic steatosis was based on the increased echogenicity of the liver on USG compared with the cortex of the right kidney, vascular blurring of the hepatic vein trunk, and deep attenuation in the right hepatic lobe. Severity of hepatic steatosis was classified as: (1) no steatosis (normal echo texture), (2) mild steatosis (mild increase in fine echoes with blurring of diaphragm and intrahepatic vessels), and (3) moderate to severe steatosis (marked increase in fine echoes with poorly visible or invisible intrahepatic vessels, diaphragm, and posterior right lobe), which has been a reliable and accurate imaging technique for screening hepatic steatosis [9].

### 2.5 Definitions of SLD Subtypes

In accordance to the Multi‐Society Consensus [5], all subjects with hepatic steatosis by imaging were diagnosed as having SLD. Among them, cardiometabolic dysfunction was determined when at least one of the CMRF was identified [5]:

1. BMI ≥ 23 kg/m^2^ (Asian definition of overweight) OR waist circumference ≥ 90 cm (males) and ≥ 80 cm (females)
2. Fasting serum glucose ≥ 100 mg/dL OR 2‐h postload glucose levels ≥ 140 mg/dL OR HbA1c ≥ 5.7% OR a previous diagnosis of Type 2 DM (T2DM) OR treatment for T2DM
3. Blood pressure ≥ 130/85 mmHg OR treatment for HTN
4. Plasma triglycerides ≥ 150 mg/dL or lipid‐lowering treatment
5. Plasma HDL‐cholesterol ≤ 40 mg/dL (males) and ≤ 50 mg/dL (females) OR lipid‐lowering treatment

For patient who had no history of other liver conditions, and absence of viral hepatitis or notable alcohol intake, the diagnosis of MASLD was made [5]. Patient who had hepatic steatosis but without any CMRF is defined as “simple SLD.”

Patients who had no hepatic steatosis were categorized as non‐SLD. Non‐SLD patients who carried any CMRF were defined as “non‐SLD/CMRF+”; non‐SLD without any CMRF were defined as “healthy control” (Figure 1).

### 2.6 The Grade and Dynamic of Cardiometabolic Dysfunction (CMD) During Follow‐Up

We categorized the grade of CMD as minimal, moderate, and significant, according to the number of CMRF presented: (1) minimal CMD, presenting zero or one CMRF; (2) moderate CMD, presenting two or three CMRFs; and (3) significant CMD, presenting four or five CMRFs.

During each follow‐up visit, the participants underwent complete anthropometric measurement and blood tests. The dynamic of CMD grade was retrieved as the CMD grade (minimal, moderate, and significant) at the time when participants developed new onset HTN or DM, or at their last visit if participants did not develop new‐onset HTN or DM.

### 2.7 Definitions of HTN and DM

HTN is defined as persistent systolic blood pressure (SBP) ≥ 140 mmHg, diastolic BP (DBP) ≥ 90 mmHg, or the use of antihypertensive agents [10]. The year of new‐onset HTN was defined as the first year in the series during which time the subjects met the definition of HTN or began using antihypertensive agents.

DM is defined as fasting glucose (FG) ≥ 126 mg/dL, hemoglobin A1C (HbA1c) ≥ 6.5%, or the use of antihyperglycemic agents [11]. The year of new‐onset DM was defined as the first year during which time the subjects met the definition of DM or began taking antihyperglycemic agents.

### 2.8 Laboratory Data

FG, aspartate aminotransferase (AST), alanine aminotransferase (ALT), low‐density lipoprotein cholesterol (LDL‐C), high‐density lipoprotein cholesterol (HDL‐C), total cholesterol and triglycerides, HBV surface antigen (HBsAg), HCV antibody (anti‐HCV) were retrieved. Body mass index (BMI) cut‐offs were suggested by the WHO guidelines for Asian (underweight < 18.5 kg/m^2^, normal weight 18.5–22.9 kg/m^2^, overweight 23–26.9 kg/m^2^, and obese ≥ 27 kg/m^2^) [12].

### 2.9 Statistical Analyses

Data were analyzed with JMP version 16 (SAS Inc., Cary, NC, USA). Cohort characteristics were described using numbers with percentage (%), and the results of continuous variables are expressed as the mean ± standard deviation. Pearson's chi‐square test was used to analyze the dichromatic differences. Odds ratios (ORs) were determined using logistic regression in the cross‐sectional cohort. Factors associated with prevalent HTN and DM [4], excluding CMRFs were further evaluated in multiple logistic regression analyses. Kaplan–Meier survival curves were used to determine the cumulative incidences and lifetime risk of new‐onset HTN and DM. Adjusted hazard ratios (aHRs) for factors associated with new‐onset HTN and DM [4], excluding CMRFs were determined using the Cox proportional hazards model. All tests were two‐tailed, and statistical significance was set at *p* < 0.05.

To validate the role of MASLD and CMRF on the risks of HTN and DM among different groups, we constructed two matched cohorts: (1) age‐, sex‐, and BMI‐matched cohorts between individuals with MASLD versus those with non‐SLD; (2) age‐, sex‐, and CMRF‐matched cohorts between individuals with MASLD versus those with non‐SLD/CMRF+ to evaluate the impact of SLD on the incidence of new‐onset HTN or DM after adjustment for CMRF.

## 3 Results

### 3.1 Cross‐Sectional Cohort

Of the 32,569 subjects in the cross‐sectional cohort (mean age: 40.3 years, 79.2% male), 54.9% of subjects had SLD, including 16,762 (93.8% of SLD; 51.4% of cross‐sectional cohort) subjects who met the criteria of MASLD and 1113 (6.2% of SLD; 3.4% of cross‐sectional cohort) subjects with simple SLD. The prevalence rates of existing HTN and DM in MASLD subjects were 20.0% and 5.3%, respectively. Of the 14,694 subjects who were non‐SLD, 9859 (67.1% of non‐SLD; 30.3% of cross‐sectional cohort) subjects had at least one CMRF, whereas 4835 (32.9% of non‐SLD; 14.8% of cross‐sectional cohort) subjects were healthy controls. The prevalence rates of existing HTN and DM in non‐SLD/CMRF+ subjects were 11.0% and 1.9%, respectively (Table 1).

**TABLE 1:** Demographic data of cross‐sectional and longitudinal cohorts.

Variables^a^	Cross‐sectional cohorts							Longitudinal cohorts						
	All (*n* = 32,569)	SLD			Non‐SLD			All (*n* = 27,109)	SLD			Non‐SLD		
		Total (*n* = 17,875)	MASLD (*n* = 16,762)	Simple SLD (*n* = 1113)	Total (*n* = 14,694)	Non‐SLD/CMRF+ (*n* = 9859)	Healthy control (*n* = 4835)		Total (*n* = 13,733)	MASLD (*n* = 12,620)	Simple SLD (*n* = 1113)	Total (*n* = 13,376)	Non‐SLD/CMRF+ (*n* = 8547)	Healthy control (*n* = 4829)
Age, years	40.3 ± 8.9	41.6 ± 8.5	41.8 ± 8.5	37.6 ± 8.5	38.8 ± 9.2	40.1 ± 9.1	36.3 ± 8.8	39.3 ± 8.8	40.3 ± 8.4	40.5 ± 8.4	37.6 ± 8.5	38.2 ± 9.0	39.3 ± 9.0	36.2 ± 8.8
Sex, male, *n* (%)	25,797 (79.2)	15,170 (84.9)	14,587 (87.0)	583 (52.4)	10,627 (72.3)	8071 (81.9)	2556 (52.9)	20,901 (77.1)	11,456 (83.4)	10,873 (86.2)	583 (52.4)	9445 (70.6)	6895 (80.7)	2550 (52.8)
Body height, cm	167.1 ± 7.4	167.5 ± 7.1	167.7 ± 7.0	164.9 ± 7.9	166.6 ± 7.6	167.3 ± 7.3	165.3 ± 8.1	167.2 ± 7.5	167.6 ± 7.2	167.9 ± 7.1	164.9 ± 7.9	166.6 ± 7.7	167.4 ± 7.4	165.3 ± 8.1
Body weight, kg	67.9 ± 11.7	72.4 ± 11.1	73.4 ± 10.7	57.9 ± 7.2	62.5 ± 10.0	65.7 ± 9.4	55.9 ± 7.5	66.9 ± 11.4	71.5 ± 10.9	72.7 ± 10.3	57.9 ± 7.2	62.1 ± 9.9	65.6 ± 9.4	55.9 ± 7.5
BMI, kg/m^2^	24.2 ± 3.4	25.7 ± 3.1	26.0 ± 3.0	21.2 ± 1.4	22.4 ± 2.7	23.4 ± 2.5	20.4 ± 1.7	23.8 ± 3.2	25.4 ± 3.0	25.7 ± 2.8	21.2 ± 1.4	22.3 ± 2.6	23.3 ± 2.5	20.4 ± 1.7
SBP, mmHg	124.7 ± 15.5	127.5 ± 15.6	128.6 ± 15.4	112.0 ± 9.8	121.2 ± 14.5	125.7 ± 14.4	111.9 ± 9.7	121.0 ± 12.4	122.9 ± 12.0	123.8 ± 11.7	112.0 ± 9.8	119.0 ± 12.4	123.0 ± 12.0	111.9 ± 9.7
DBP, mmHg	79.5 ± 11.0	81.8 ± 11.1	82.5 ± 11.0	71.1 ± 7.4	76.7 ± 10.3	79.5 ± 10.4	70.9 ± 7.1	77.0 ± 9.1	78.7 ± 8.9	79.3 ± 8.7	71.1 ± 7.4	75.3 ± 9.0	77.7 ± 9.0	70.9 ± 7.1
Smoker, *n* (%)	6406 (19.7)	3823 (21.4)	3650 (21.8)	173 (15.6)	2583 (17.6)	1870 (19.0)	713 (14.7)	5352 (19.7)	2999 (21.8)	2826 (22.4)	173 (15.6)	2353 (17.6)	1641 (19.2)	712 (14.7)
Fasting blood sugar, mg/dL	96.1 ± 21.7	99.2 ± 24.5	99.9 ± 25.2	89.3 ± 6.6	92.3 ± 16.8	94.6 ± 19.4	87.4 ± 7.1	92.6 ± 10.9	94.5 ± 11.3	94.9 ± 11.5	89.3 ± 6.6	90.7 ± 10.1	92.5 ± 11.0	87.4 ± 7.1
Total cholesterol,^b^ mg/dL	188.0 ± 33.4	193.0 ± 33.8	194.0 ± 33.8	178.2 ± 29.7	182.0 ± 31.9	184.7 ± 32.6	176.5 ± 29.9	186.2 ± 32.7	191.1 ± 32.9	192.3 ± 32.9	178.2 ± 29.7	181.2 ± 31.7	183.8 ± 32.4	176.5 ± 30.0
Triglyceride,^c^ mg/dL	133.4 ± 100.7	162.3 ± 117.1	167.6 ± 118.9	82.6 ± 28.9	98.3 ± 59.3	110.2 ± 66.9	73.9 ± 26.1	124.0 ± 85.8	151.9 ± 99.7	158.0 ± 101.4	82.6 ± 28.9	95.4 ± 55.5	107.6 ± 63.5	73.9 ± 26.1
HDL‐C,^d^ mg/dL	54.4 ± 13.2	50.2 ± 11.4	49.3 ± 10.9	60.9 ± 12.4	59.3 ± 13.5	56.0 ± 13.1	64.4 ± 12.3	55.1 ± 13.3	50.7 ± 11.6	49.6 ± 10.9	60.9 ± 12.4	59.4	55.9	64.4
												±13.5	±13.1	±12.3
LDL‐C,^e^ mg/dL	110.8 ± 31.6	114.2 ± 32.0	115.0 ± 32.2	103.9 ± 27.6	106.9 ± 30.6	110.6 ± 31.1	101.1 ± 28.9	109.8 ± 30.6	113.5 ± 30.6	114.5 ± 30.7	103.9 ± 27.6	106.2	109.9	101.1
												±30.3	±30.7	±28.9
AST, U/L	17.4 ± 9.6	18.7 ± 10.9	19.0 ± 11.0	14.9 ± 6.6	15.8 ± 7.6	16.1 ± 8.3	15.0 ± 5.8	16.9 ± 8.3	18.1 ± 9.4	18.4 ± 9.6	14.9 ± 6.6	15.6	16.0	15.0
												±6.7	±7.2	±5.8
ALT, U/L	19.1 ± 16.1	23.2 ± 18.9	23.8 ± 19.1	13.9 ± 10.0	14.2 ± 9.9	15.1 ± 10.5	12.3 ± 8.4	18.3 ± 15.1	22.4 ± 18.2	23.1 ± 18.6	13.9 ± 10.0	14.0	15.0	12.3
												±9.4	±9.8	±8.4
Hypertension, *n* (%)	4437 (13.6)	3352 (18.8)	3352 (20.0)	0 (0.0)	1085 (7.4)	1085 (11.0)	0 (0.0)	0 (0.0)	0 (0.0)	0 (0.0)	0 (0.0)	0 (0.0)	0 (0.0)	0 (0.0)
Diabetes, *n* (%)	1079 (3.3)	891 (5.0)	891 (5.3)	0 (0.0)	188 (1.3)	188 (1.9)	0 (0.0)	0 (0.0)	0 (0.0)	0 (0.0)	0 (0.0)	0 (0.0)	0 (0.0)	0 (0.0)

Abbreviations: ALT: alanine aminotransferase; AST: aspartate aminotransferase; BMI: body mass index; CMRF: cardiometabolic risk factor; DBP: diastolic blood pressure; HDL‐C: high‐density lipoprotein cholesterol; LDL‐C: low‐density lipoprotein cholesterol; MASLD: metabolic dysfunction‐associated steatotic liver disease; SBP: systolic blood pressure; SLD: steatotic liver disease.

^a^ Data are shown as the number (%) or means ± standard deviations unless otherwise indicated.

^b^ Missing data *n* = 18 in cross‐sectional cohorts, *n* = 17 in longitudinal cohorts.

^c^ Missing data *n* = 41 in cross‐sectional cohorts, *n* = 30 in longitudinal cohorts.

^d^ Data were based on 9279 in cross‐sectional cohorts and 8007 subjects in longitudinal cohorts.

^e^ Data were based on 9266 in cross‐sectional cohorts and 8001 subjects in longitudinal cohorts.

### 3.2 Factors Associated With Existing HTN/DM in the Cross‐Sectional Cohort

In the cross‐sectional cohort, the prevalence of HTN was 7.38%, 11.00%, and 20.00% among subjects with non‐SLD, non‐SLD/CMRF+, and MASLD, respectively, increasing with the grade of CMD (1.51%, 20.75%, and 40.66% for minimal, moderate, and significant CMD). Multivariate regression, adjusted for age, sex, smoking, AST, and ALT levels, showed MASLD had significantly higher HTN prevalence compared to non‐SLD (aOR/95% CI: 2.40/2.23–2.59) and non‐SLD/CMRF+ (aOR/95% CI: 1.74/1.61–1.88). Moderate and significant CMD were linked to higher HTN rates compared to minimal CMD (aOR/95% CI: 14.17/11.10–18.08 and 32.67/25.37–42.08) (Table S1).

Similarly, DM prevalence was 1.28%, 1.91%, and 5.32% among subjects with non‐SLD, non‐SLD/CMRF+, and MASLD, respectively, increasing with CMD grade (0.44%, 3.86%, and 17.50% for minimal, moderate, and significant CMD). MASLD had higher DM rates compared to non‐SLD (aOR/95% CI: 3.17/2.69–3.73) and non‐SLD/CMRF+ (aOR/95% CI: 2.39/2.03–2.81). Moderate and significant CMD were also associated with higher DM rates compared to minimal CMD (aOR/95% CI: 7.27/4.62–11.44 and 32.20/20.42–50.79) (Table S2).

### 3.3 Longitudinal Cohort

Of 27,109 subjects in the longitudinal cohort (mean age, 39.3 years; 77.1% male, Table 1), the mean follow‐up period was 6.28 (± 4.15) years (range, 1–14 years) in assessing HTN development, and 6.58 (± 4.29) years (range, 1–14 years) in assessing DM development.

### 3.4 Incidence Rate of New‐Onset HTN

Of 27,109 subjects, 2358 subjects developed new‐onset HTN during 170,378 person‐years follow‐up (annual incidence, 13.8/1000 person‐years) The 3‐, 5‐, 7‐, and 10‐year cumulative incidences of HTN were 2.49%, 5.34%, 8.68%, and 14.10%, respectively. The cumulative lifetime risk of HTN at 40, 50, 60, and 65 years old was 1.31%, 8.31%, 23.49%, and 32.70%, respectively (Table S4).

### 3.5 Risk of New‐Onset HTN Among Different Subgroups

Among 13,733 SLD subjects, 91.90% had MASLD. Among MASLD subjects, 1599 (12.67%) developed HTN during 81,184 person‐years follow‐up (annual incidence, 19.7/1000 person‐years). By contrast, 16 (1.44%) subjects with simple SLD developed HTN during 6704 person‐years follow‐up (annual incidence, 2.4/1000 person‐years) (Table 2). 704 (8.24%) developed HTN during 54,764 person‐years follow‐up (annual incidence, 12.9/1000 person‐years) and 39 (0.81%) developed HTN during 27,726 person‐years follow‐up (annual incidence, 1.4/1000 person‐years) among non‐SLD/CRMF+ subjects and healthy controls, respectively.

**TABLE 2:** Subgroup risk of new‐onset of HTN and DM in a longitudinal cohort.

	New‐onset HTN/DM			Cox proportional regression							
	Total, *n*	Yes, *n* (%)	Annual incidence (per 1000 person‐years)	Univariate HRs (95% CI)	*p*	Multivariate HRs^a^ (95% CI)	*p*	Multivariate HRs^a^ (95% CI)	*p*	Multivariate HRs^a^ (95% CI)	*p*
*HTN*											
MASLD											
Non‐SLD	13,376	743 (5.55)	9.0	1		1					
MASLD	12,620	1599 (12.67)	19.7	2.17 (1.99–2.37)	**< 0.0001**.	1.89 (1.73–2.07)	**< 0.0001**				
Comparison among subgroups											
Healthy control	4829	39 (0.81)	1.4	1		1					
Non‐SLD/CMRF+	8547	704 (8.24)	12.9	8.85 (6.41–12.22)	**< 0.0001**	7.48 (5.41–10.34)	**< 0.0001**	1			
Simple SLD	1113	16 (1.44)	2.4	1.69 (0.94–3.02)	0.0782	1.66 (0.93–2.97)	0.0878	0.22 (0.14–0.36)	**< 0.0001**	1	
MASLD	12,620	1599 (12.67)	19.7	13.59 (9.89–18.68)	**< 0.0001**	10.71 (7.78–14.74)	**< 0.0001**	1.43 (1.31–1.57)	**< 0.0001**	6.45 (3.94–10.57)	**< 0.0001**
*DM*											
MASLD											
Non‐SLD	13,376	54 (0.40)	0.6	1		1					
MASLD	12,620	545 (4.32)	6.3	9.87 (7.47–13.06)	**< 0.0001**	8.24 (6.22–10.92)	**< 0.0001**				
Comparison among subgroups											
Healthy control	4829	2 (0.04)	0.1	1		1					
Non‐SLD/CMRF+	8547	52 (0.61)	0.9	12.00 (2.92–49.27)	**0.0006**	9.66 (2.35–39.71)	**0.0017**	1			
Simple SLD	1113	2 (0.18)	0.3	4.07 (0.57–28.90)	0.1604	3.93 (0.55–27.92)	0.1710	0.41 (0.10–1.67)	0.2125	1	
MASLD	12,620	545 (4.32)	6.3	84.18 (21.00–337.46)	**< 0.0001**	60.49 (15.07–242.87)	**< 0.0001**	6.26 (4.70–8.33)	**< 0.0001**	15.38 (3.83–61.78)	**< 0.0001**

*Note*: Bold values indicates statistical significance, *p* < 0.05.

Abbreviations: CI: confidence interval; CMRF: cardiometabolic risk factor; DM: diabetes; HR: hazard ratio; HTN: hypertension; MASLD: metabolic dysfunction‐associated steatotic liver disease; SLD: steatotic liver disease.

^a^ Adjusted for age; sex; smoking; and AST and ALT levels.

**FIGURE 2:**
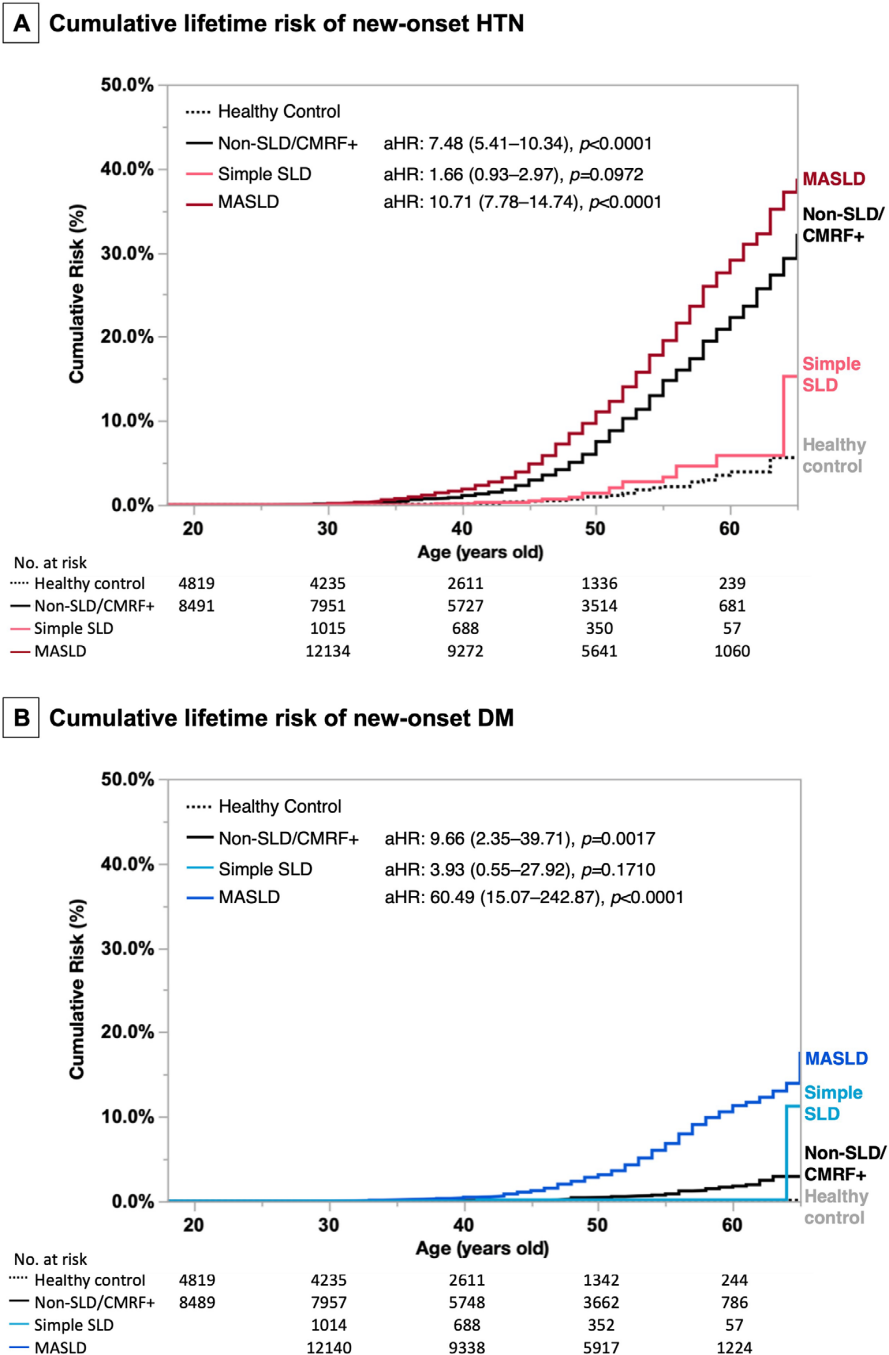
Cumulative lifetime risk of new‐onset hypertension and diabetes. (A) New‐onset hypertension and (B) new‐onset diabetes.

In Kaplan–Meier analysis, the 3‐, 5‐, 7‐, and 10‐year cumulative incidences of HTN were 0.18%, 0.34%, 0.68%, and 1.08% in healthy controls, compared to 3.68%, 7.72%, 12.25%, and 17.32%, respectively, in MASLD subjects (cHR/CI: 13.59/9.89–18.68, *p* < 0.0001). The cumulative lifetime risk of HTN at 40, 50, 60, and 65 years old was 0.16%, 0.95%, 3.92%, and 5.60%, respectively, in the healthy control group, compared to 1.87%, 11.06%, 29.09%, and 38.79%, respectively, in MASLD subjects (cHR/CI: 10.01/7.28–13.75, *p* < 0.0001; Figure 2A, Tables S52 and S6).

### 3.6 Impact of MASLD and CMRF on New‐Onset HTN

In Cox proportional regression analyses, MASLD subjects remained at significantly higher risk of new‐onset HTN, compared to those with non‐SLD (aHR/95% CI: 1.89/1.73–2.07, Table 2). Consistent results were observed in the analysis with age‐, sex‐, and BMI‐matched cohorts (Table S7). Compared to those without CMRF, the presence of CMRF increased the risk of new‐onset HTN in both SLD (MASLD vs. simple SLD, aHR/95% CIs: 6.45/3.94–10.57) and non‐SLD patients (non‐SLD/CMRF+ vs. healthy control, aHR/95% CIs: 7.48/5.41–10.34, Table 2). For patients with CMRF, MASLD increased the risk of new‐onset HTN more than in patients without SLD (non‐SLD/CMRF+, aHR/95% CI: 1.43/1.31–1.57). However, the difference in risk of new‐onset HTN no longer existed between MASLD and non‐SLD/CMRF+ subjects in the age‐, sex‐, CMRF‐matched cohorts (Table S8).

Compared to healthy controls, significantly higher risks of new‐onset HTN was observed in subjects with MASLD, but not in simple SLD subjects (Table 2).

### 3.7 Number of Fulfilled CMRF of MASLD and New‐Onset HTN

Among the 13,733 subjects with SLD in the longitudinal cohort, 26.51%, 36.70%, 26.70%, 9.28%, and 0.81% had one, two, three, four, and five CMRFs, respectively. The annual incidence of new‐onset HTN was 5.4, 17.1, 29.4, 42.6, and 40.8 per 1000 person‐years for subjects with one to five CMRFs, respectively (Table S9).

**FIGURE 3:**
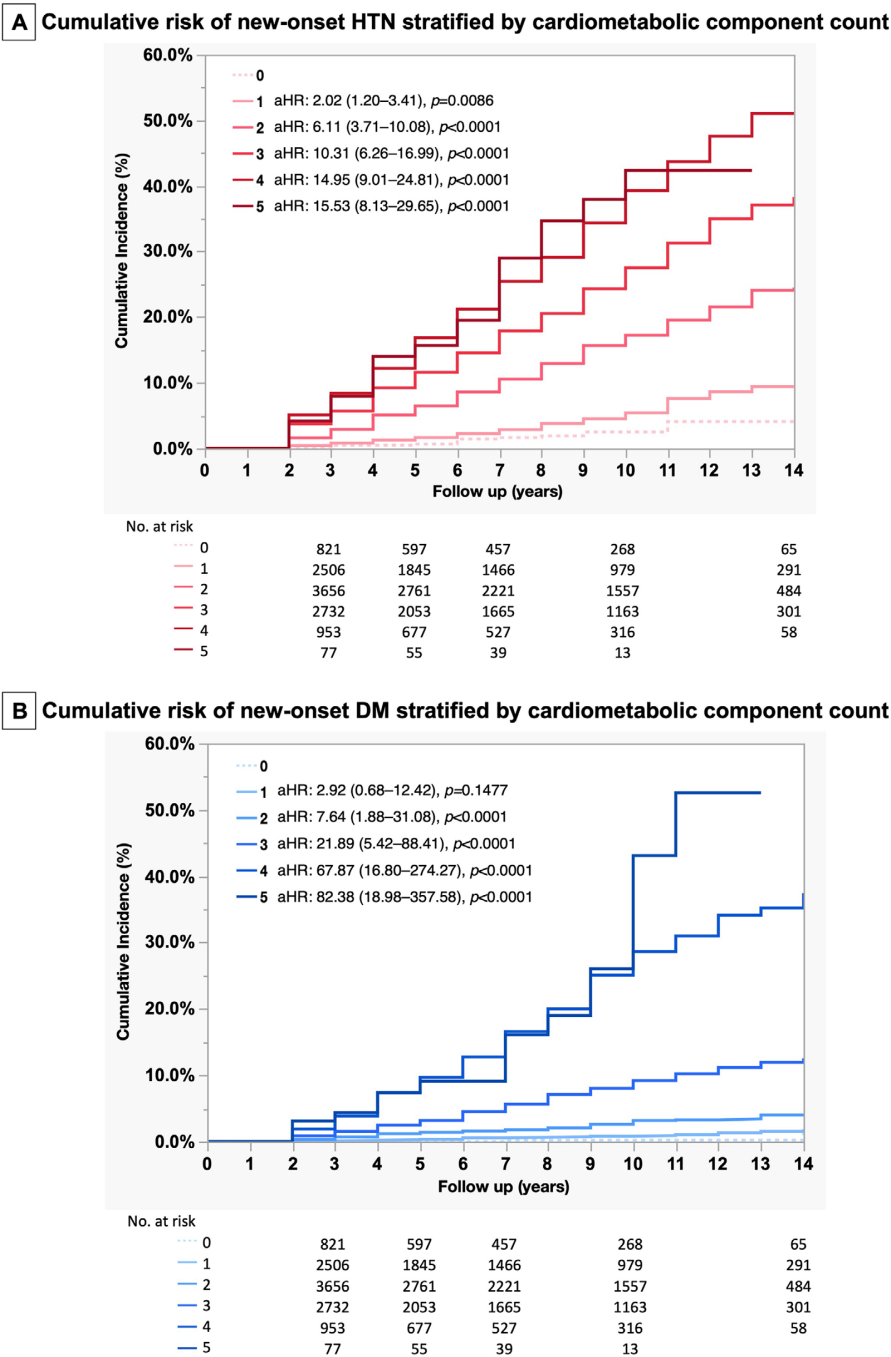
Cumulative incidence of new‐onset hypertension and diabetes by the number of cardiometabolic components of SLD. (A) New‐onset hypertension and (B) new‐onset diabetes.

In Cox proportional regression analyses, the risk of new‐onset HTN increased robustly with the fulfilled number of CMRF when compared to those without any CMRF (aHR/CI from 2.02/1.20 to 3.41 in one‐CMRF subjects to 15.53/8.13–29.65 in five‐CMRFs subjects, Figure 3A).

### 3.8 Dynamic in CMD Grade and Risk of New‐Onset HTN Among SLD Subjects During Follow‐Up

**FIGURE 4:**
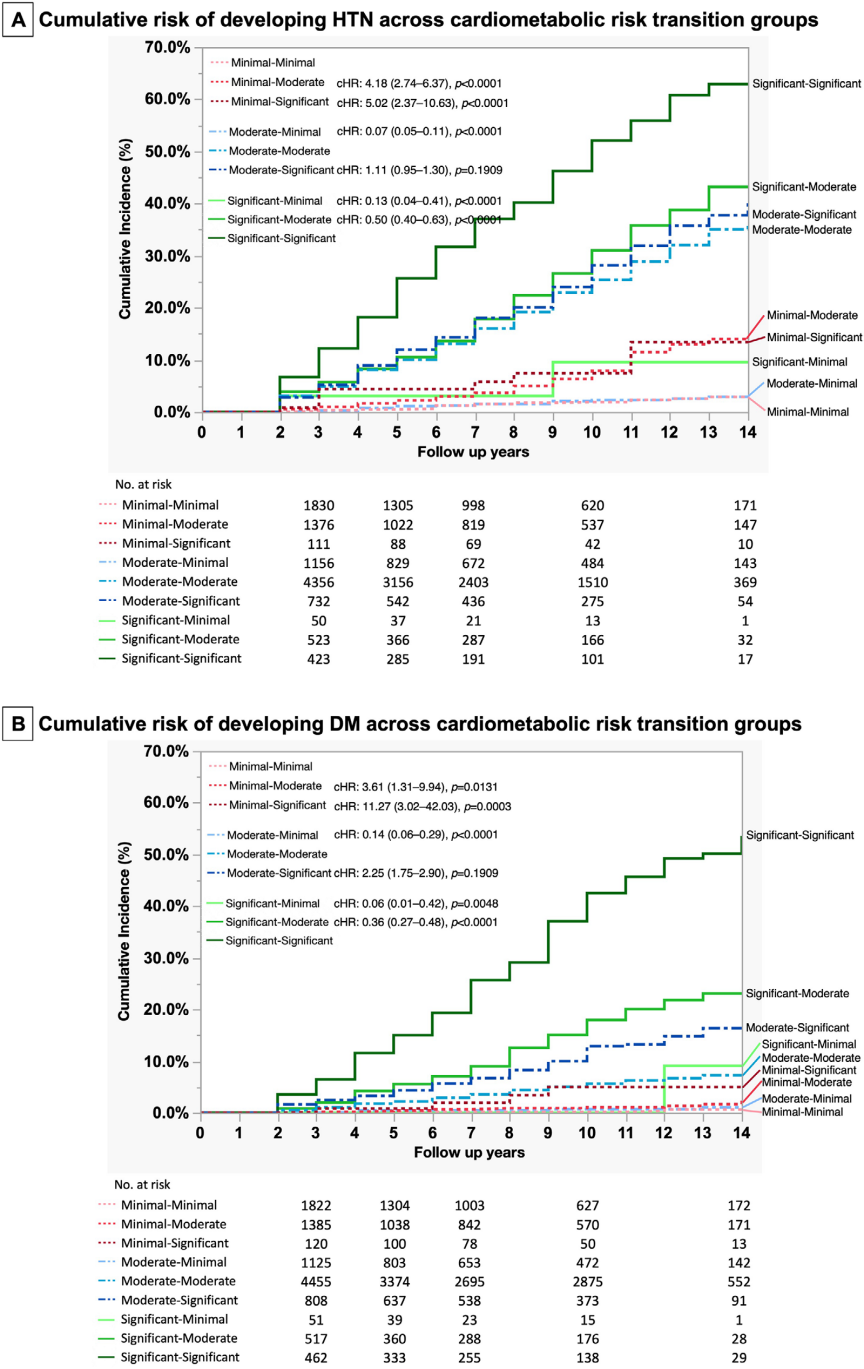
Cumulative incidence of new‐onset hypertension and diabetes across cardiometabolic risk transition groups among SLD patients. (A) New‐onset hypertension and (B) new‐onset diabetes.

Among 4459 subjects with minimal CMD during the first visit, 39.45% progressed to moderate CMD and 2.83% to significant CMD at the censored date or last visit. The annual incidence of new‐onset HTN was 1.8/1000 person‐years for subjects with persistent minimal CMD, 8.0/1000 person‐years for subjects progressing to moderate CMD, and 9.7/1000 person‐years for those progressing to significant CMD. Cox proportional regression analyses showed that the risk of new‐onset HTN was significantly higher in those progressing to moderate CMD (cHR/CI: 4.18/2.74–6.73) and significant CMD (cHR/CI: 5.02/2.37–10.63) compared to those who remained minimal CMD (Table S10 and Figure 4A).

Among 8001 subjects with moderate CMD at the first visit, 18.39% regressed to minimal CMD at the end‐of‐follow‐up and 11.46% progressed to significant CMD. The annual incidence of HTN was 2.0/1000 person‐years for those regressing to minimal CMD, 26.6/1000 person‐years for subjects with persistent moderate CMD, and 29.7/1000 person‐years for those progressing to significant CMD. Cox proportional regression analysis showed that subjects with CMD regressing to minimal CMD had significantly lower risks of new‐onset HTN (cHR/CI: 0.07/0.05–0.11) compared to those who remained with moderate CMD.

Among 1273 subjects with significant CMD at the first visit, 5.26% regressed to minimal CMD at the end of follow‐up and 52.24% to moderate CMD at the censored date or last visit. The annual incidence of HTN was 7.9/1000 person‐years for subjects regressing to minimal CMD, 31.8/1000 person‐years for subjects regressing to moderate CMD, and 60.8/1000 person‐years for those with persistent significant CMD. Cox proportional regression analysis showed that subjects with CMD regressing to minimal and moderate CMD had significantly lower risks of new‐onset HTN (cHR/CI: 0.13/0.04–0.41 and 0.50/0.40–0.63, respectively) compared to those who remained with significant CMD.

### 3.9 Incidence and Risk of New‐Onset DM

A total of 601 subjects developed new‐onset DM during 178,255 person‐years follow‐up (annual incidence, 3.4/1000 person‐years). The 3‐, 5‐, 7‐, and 10‐year cumulative incidences of DM were 0.59%, 1.34%, 2.26%, and 3.82%, respectively. The cumulative lifetime risk of DM at 40, 50, 60, and 65 years old was 0.29%, 1.79%, 6.70%, and 10.90%, respectively (Table S4).

### 3.10 Risk of New‐Onset DM and SLD

Among MASLD subjects, 545 (4.32%) developed DM over 86,042 person‐years (annual incidence 6.3/1000 person‐years). In contrast, only 2 (0.18%) of simple SLD subjects developed DM over 6743 person‐years (annual incidence 0.3/1000 person‐years). Among non‐SLD/CMRF+ subjects, 52 (0.61%) developed DM over 57,657 person‐years (annual incidence 0.9/1000 person‐years), while only 2 (0.04%) of healthy controls developed DM over 27,813 person‐years (annual incidence 0.1/1000 person‐years) (Table 2).

Kaplan–Meier analysis showed that the 3‐, 5‐, 7‐, and 10‐year cumulative incidences of DM were significantly higher in MASLD subjects (1.13%, 2.50%, 4.13%, and 7.08%) compared to healthy controls (0.00%, 0.08%, 0.08%, and 0.08%) (cHR/95% CI: 84.55/21.09–338.95, *p* < 0.0001). The cumulative lifetime risk of DM at ages 40, 50, 60, and 65 was also higher in MASLD subjects (0.47%, 3.13%, 11.31%, and 17.69%) compared to healthy controls (0.00%, 0.10%, 0.10%, and 0.10%) (cHR/95% CI: 62.11/15.49–248.98, *p* < 0.0001) (Figure 2B, Tables S11 and S12).

### 3.11 Impact of MASLD and CMRF on New‐Onset DM

In Cox proportional regression analyses, MASLD subjects remained at significantly higher risk of new‐onset DM compared to those with non‐SLD (aHR/95% CI: 8.24/6.22–10.92, Table 2). Consistent results were observed in the analysis with age‐, sex‐, BMI‐matched cohorts (Table S7). Compared to those without CMRF, the presence of CMRF increased the risk of new‐onset DM in both SLD (MASLD vs. simple SLD, aHR/95% CI: 15.38/3.83–61.78) and non‐SLD patients (non‐SLD/CMRF+ vs. healthy control, aHR/95% CI: 9.66/2.35–39.71, Table 2). For patients with CMRF, the presence of hepatic steatosis (MASLD) increased the risk of new‐onset DM in patients without SLD (non‐SLD/CMRF+, aHR/95% CI: 6.26/4.70–8.33). Consistent results were observed in the age‐, sex‐, CMRF‐matched cohorts (Table S8).

Compared to healthy controls, significantly higher risk of new‐onset DM was observed in MASLD subjects, but not in simple SLD subjects (Table 2).

### 3.12 Number of Fulfilled CMRF of MASLD and New‐Onset DM

Among simple SLD individuals, 2 (0.18%) developed DM during 6743 person‐years (annual incidence: 0.3/1000 person‐years). For those with one to five CMRFs, the annual incidence rates were 1.0, 2.8, 8.7, 27.5, and 30.6 per 1000 person‐years, respectively (Table S9).

In Cox proportional regression analyses, risk of new‐onset DM increased robustly with the fulfilled number of CMRF when compared to those without any CMRF (aHR/CI from 2.92/0.68–12.42 in one‐CMRF subjects to 82.38/18.98–357.58 in five‐CMRFs subjects, Figure 3B).

### 3.13 Dynamic in CMD Grade and Risk of New‐Onset DM Among SLD Subjects During Follow‐Up

Among 4459 subjects with minimal CMD during the first visit, 39.49% progressed to moderate CMD and 3.01% to significant CMD at censored date or last visit. The annual incidence of new‐onset DM was 0.3/1000 person‐years for subjects with persistent minimal CMD, 1.2/1000 person‐years for subjects progressing to moderate CMD and 3.8/1000 person‐years for those progressing to significant CMD. Cox proportional regression analyses showed that the risk of new‐onset DM was significantly higher in those progressing to moderate CMD (cHR/CI: 3.61/1.31–9.94) and significant CMD (cHR/CI: 3.61/1.31–9.94) compared to those who remained with minimal CMD (Table S13 and Figure 4B).

Among 8001 subjects with moderate CMD at the first visit, 17.95% regressed to minimal CMD at the end‐of‐follow‐up and 12.29% progressed to significant CMD. The annual incidence of DM was 0.7/1000 person‐years for those regressing to minimal CMD, 5.2/1000 person‐years for subjects with persistent moderate CMD, and 11.9/1000 person‐years for those progressing to significant CMD. Cox proportional regression analysis showed that subjects regressing to minimal CMD had lower risk of new‐onset DM (cHR/CI: 0.14/0.06–0.29), while those progressing to significant CMD had higher risk (cHR/CI: 2.25/1.75–2.90) compared to those remained with moderate CMD.

Among 1273 subjects with significant CMD at the first visit, 5.11% regressed to minimal CMD at the end‐of‐follow‐up and 50.35% to moderate CMD at censored date or last visit. The annual incidence of DM was 2.5/1000 person‐years for those regressing to minimal CMD subjects, 15.9/1000 person‐years for subjects regressing to moderate CMD, and 43.6/1000 person‐years for those with persistent significant CMD. Cox proportional regression analysis showed that subjects with CMD regressing to minimal and moderate CMD had significantly lower risks of new‐onset DM (cHR/CI: 0.06/0.01–0.42 and 0.36/0.27–0.48, respectively) compared to those who remained with significant CMD.

## 4 Discussion

In this large‐scale study, we demonstrated that patients with MASLD are at high risks of HTN and DM in both cross‐sectional and longitudinal settings. The prevalence of HTN and DM was 7.38% and 1.28% among subjects with non‐SLD, compared to 20.00% and 5.32% among those with MASLD, respectively. The annual incidences of incident HTN and DM per 1000 person‐years were higher among MASLD subjects (19.7 and 6.3) compared to healthy controls (1.4 and 0.1), non‐SLD/CMRF+ (12.9 and 0.9), and simple SLD (2.4 and 0.3), respectively.

In the current study, MASLD was correlated with a 2.40‐ and 3.17‐times higher odds of pre‐existing HTN and DM, respectively, and a 1.89‐ and 8.24‐fold risk of developing new‐onset HTN and DM, respectively, compared to patients with non‐SLD. For patients with CMRF, the presence of hepatic steatosis (MASLD) was associated with 1.74‐ and 2.39‐times higher odds of pre‐existing HTN and DM, respectively, and a 1.43‐ and 6.26‐fold risk of developing new‐onset HTN and DM, respectively, compared to patients without SLD (non‐SLD/CMRF+).

We also observed that the presence of CMRF significantly increased the risk of developing HTN and DM, with a 6.45‐ and 15.38‐fold risk in SLD patients, and a 7.48‐ and 9.66‐fold risk in non‐SLD patients. Moreover, the incidence of new‐onset HTN and DM increased with the increasing number of CMRF in MASLD, with a 2.02‐ and 2.92‐fold risk in one‐CMRF subjects, and a 15.53‐ and 82.38‐fold risk in five‐CMRF subjects. SLD and chronic metabolic disorders, such as T2DM and HTN, share common pathophysiological mechanisms, including insulin resistance and chronic inflammation, which ultimately contribute to the development of atherosclerosis and CVD [13]. As a result, screening for metabolic dysfunction in individuals with SLD allows the early identification of HTN and DM risk, which could help in advising proactive lifestyle modification to prevent HTN and DM development [14, 15].

In our previous study, we found that development or progression of SLD are significantly associated with increased risks of developing HTN and DM; by contrast, regression or resolved SLD are associated with reduced risks of developing HTN and DM [4]. Similarly, we also observed a significant impact of the dynamic changes in CMD grade during follow‐up on the risk of HTN and DM. The progression of CMD is significantly associated with increased risks of developing HTN and DM. Conversely, CMD regression is associated with reduced risks of developing HTN and DM. These findings suggest the importance of monitoring and managing CMRF, including body weight, blood pressure, blood sugar, and lipid profiles, as potential risk factors for HTN and DM.

The prevalence of MASLD was 51.5% in cross‐sectional and 46.6% in longitudinal which is higher than previous studies in the United States (18.7%–33.4%) [16, 17] and at global spectrum (around 30%) [18, 19]. This may result from older age and male‐dominant in our cohorts. In the current study, an estimated 1.9‐ and 8.2‐fold risk of HTN and diabetes when compared with non‐SLD patients. Supporting these data is a recent meta‐analysis focusing on longitudinal outcomes associated with MASLD revealed a significant increase risk of as incident HTN (HR, 1.75) and diabetes (HR, 2.56) among MASLD patients compared with non‐MASLD individuals [18]. Another large‐scale study on different subtypes of SLD and diabetes revealed that MASLD (HR, 6.64) was independently associated with an increased risk of diabetes which further supports the conclusion that MASLD are essential drivers of diabetes [20]. Notably, we found that simple SLD did not increase the risk of HTN and DM, as observed by He et al. [20] that cryptogenic SLD (simple SLD) were not associated with diabetes risk.

Comparing to our previous research on NAFLD [4], MASLD as the new terminology of FLD, showed similar risks of new HTN and diabetes. This, thereby raises the importance of managing CMRF alongside addressing the severity of FL. It is noteworthy that the cardiometabolic criteria purposed by the Delphi consensus included stricter threshold for blood pressure (≥ 130/85 mmHg) and FG (≥ 100 mg/dL), which are recognized as pre‐HTN and pre‐DM [5]. These findings align with our previous study [4], which concluded that both pre‐HTN and pre‐DM are significant risk factors for the prevalence and incidence of HTN and DM.

There were some limitations in the current study. First, around 70% of the subjects had missing HDL lab data, which could lead to an underestimation of the prevalence of MASLD and its potential impact on the incidence of HTN and DM. However, because the diagnosis of MASLD is established by meeting at least one of the five cardiometabolic criteria, the effect of the absent data would primarily apply to patients lacking any CMRFs in this context. Second, all MASLD patients were advised to execute life style modification, exercise and diet control. However, we were unable to obtain the data regarding the adherence of the advice among the participants. Third, due to the 2017 ACC/AHA guidelines lowering the HTN threshold from an SBP of 140 to 130 mmHg [21], the target population for achieving HTN outcomes may be affected.

In conclusion, MASLD is a significant risk factor for both prevalent and incidental HTN and DM. This association becomes even more pronounced with an increased burden of CMRFs. Dynamic of CMD grade had further impact on the risks of new‐onset HTN and DM. Early intervention for metabolic dysfunction with lifestyle modification should be mandatory for SLD patients to prevent major cardiovascular and diabetic events [22, 23]. MASLD underscores the role of systemic metabolic dysregulation in driving liver disease. This will allow a clearer understanding of SLD nature, pathogenesis, and management globally and can help increase disease awareness and management.

## Disclosure

Ming‐Lung Yu has received research support from Abbvie, Abbott, BMS, Gilead, Merck, and Roche diagnostics; served as a consultant of Abbvie, Abbott, BMS, Gilead, Merck, Novartis, Roche, and Roche diagnostics; and served as a speaker of Abbvie, Abbott, BMS, Gilead, Merck, Roche, and Roche diagnostics.

## Conflicts of Interest

The authors declare no conflicts of interest.

## Supporting information


**Table S1.** Factors associated with existing HTN in the cross‐sectional cohort.
**Table S2.** Factors associated with existing DM in the cross‐sectional cohort.
**Table S3.** Correlation between the number of cardiometabolic components of MASLD and existing HTN and DM in the cross‐sectional cohort.
**Table S4.** Cumulative lifetime risk and incidence of new‐onset HTN and DM in the longitudinal cohort.
**Table S5.** Cumulative incidence of new‐onset HTN.
**Table S6.** Cumulative lifetime risk of new‐onset HTN.
**Table S7.** Age‐, sex‐, and BMI‐matched cohorts of MASLD versus non‐SLD.
**Table S8.** Age‐, sex‐, and CMRF‐matched cohorts of MASLD versus non‐SLD/CMRF+.
**Table S9.** Correlation between the number of CMRF and the incidence of new‐onset HTN and DM in the longitudinal cohort.
**Table S10.** New‐onset HTN in longitudinal cohorts across cardiometabolic risk transition groups.
**Table S11.** Cumulative incidence of new‐onset DM.
**Table S12.** Cumulative lifetime risk of new‐onset DM.
**Table S13.** New‐onset DM in longitudinal cohorts across cardiometabolic risk transition groups.
**Table S14.** Cumulative incidence of new‐onset HTN and DM across cardiometabolic risk groups.
**Table S15.** Cumulative incidence of new‐onset HTN and DM across cardiometabolic risk transition groups.

## Data Availability

Original data generated and analyzed during this study are included in this published article or in the data repositories listed in the References.
