# Supplementary material for: Dynamics of Cardiometabolic Risk Factors Are Linked to the Risk of Hypertension and Diabetes in MASLD
Source: Kaohsiung J Med Sci. 2025 Jul 12;41(12):e70077. doi: 10.1002/kjm2.70077 (PMC12694567; doi:10.1002/kjm2.70077)
Supplement: Supplementary file 1 — Table S1. Factors associated with existing HTN in the cross‐sectional cohort. Table S2. Factors associated with existing DM in the cross‐sectional cohort. Table S3. Correlation between the number of cardiometabolic components of MASLD and existing HTN and DM in the cross‐sectional cohort. Table S4. Cumulative lifetime risk and incidence of new‐onset HTN and DM in the longitudinal cohort. Table S5. Cumulative incidence of new‐onset HTN. Table S6. Cumulative lifetime risk of new‐onset HTN. Table S7. Age‐, sex‐, and BMI‐matched cohorts of MASLD versus non‐SLD. Table S8. Age‐, sex‐, and CMRF‐matched cohorts of MASLD versus non‐SLD/CMRF+. Table S9. Correlation between the number of CMRF and the incidence of new‐onset HTN and DM in the longitudinal cohort. Table S10. New‐onset HTN in longitudinal cohorts across cardiometabolic risk transition groups. Table S11. Cumulative incidence of new‐onset DM. Table S12. Cumulative lifetime risk of new‐onset DM. Table S13. New‐onset DM in longitudinal cohorts across cardiometabolic risk transition groups. Table S14. Cumulative incidence of new‐onset HTN and DM across cardiometabolic risk groups. Table S15. Cumulative incidence of new‐onset HTN and DM across cardiometabolic risk transition groups. [file KJM2-41-e70077-s001.docx]

**Supplementary Material**

**Dynamics of cardiometabolic risk factors are linked to the risk of hypertension and diabetes in MASLD**

**Supplementary Table 1.** Factors associated with existing HTN in the cross-sectional cohort

**Supplementary Table 2.** Factors associated with existing DM in the cross-sectional cohort

**Supplementary Table 3.** Correlation between the number of cardiometabolic components of MASLD and existing HTN and DM in the cross-sectional cohort

**Supplementary Table 4.** Cumulative lifetime risk and incidence of new-onset HTN and DM in the longitudinal cohort

**Supplementary Table 5.** Cumulative incidence of new-onset HTN

**Supplementary Table 6.** Cumulative lifetime risk of new-onset HTN

**Supplementary Table 7.** Age-, sex-, and BMI-matched cohorts of MASLD vs non-SLD

**Supplementary Table 8.** Age-, sex-, and CMRF-matched cohorts of MASLD vs Non-SLD/CMRF+

**Supplementary Table 9.** Correlation between the number of CMRF and incidence of new-onset HTN and DM in the longitudinal cohort

**Supplementary Table 10.** New-onset HTN in longitudinal cohorts across cardiometabolic risk transition groups

**Supplementary Table 11.** Cumulative incidence of new-onset DM

**Supplementary Table 12.** Cumulative lifetime risk of new-onset DM

**Supplementary Table 13.** New-onset DM in longitudinal cohorts across cardiometabolic risk transition groups

**Supplementary Table 14.** Cumulative incidence of new-onset HTN and DM across cardiometabolic risk groups

**Supplementary Table 15.** Cumulative incidence of new-onset HTN and DM across cardiometabolic risk transition groups

This supplementary material has been provided by the authors to give readers additional information about their work.

| **Supplementary Table 1. Factors associated with existing HTN in the cross-sectional cohort** | | | | | | |
| --- | --- | --- | --- | --- | --- | --- |
|  | **Number** | | **Logistic regression** | | | |
|  | **Total, n** | **HTN, n (%)** | **Univariate** | | **Multivariate^a^** | |
|  |  |  | **ORs (95% C.I.)** | ***p*-value** | **ORs (95% C.I.)** | ***p*-value** |
| **MASLD** |  |  |  |  |  |  |
| Non-SLD | 14694 | 1085 (7.38) | 1 |  | 1 |  |
| MASLD | 16762 | 3352 (20.00) | 3.14 (2.92–3.37) | **<0.0001** | 2.40 (2.23–2.59) | **<0.0001** |
| **Comparison among subgroups** | |  |  |  |  |  |
| Healthy control | 4835 | 0 (0.00) | - |  | - |  |
| Non-SLD/CMRF+ | 9859 | 1085 (11.00) | 1 |  | 1 |  |
| Simple SLD | 1113 | 0 (0.00) | - |  | - |  |
| MASLD | 16762 | 3352 (20.00) | 2.02 (1.88–2.18) | **<0.0001** | 1.74 (1.61–1.88) | **<0.0001** |
| **Grade of CMD in SLD^b^** |  |  |  |  |  |  |
| Minimal | 4564 | 69 (1.51) | 1 |  | 1 |  |
| Moderate | 10694 | 2219 (20.75) | 17.06 (13.39–21.73) | **<0.0001** | 14.17 (11.10–18.08) | **<0.0001** |
| Significant | 2617 | 1064 (40.66) | 44.63 (34.75–57.32) | **<0.0001** | 32.67 (25.37–42.08) | **<0.0001** |
| Abbreviations: MASLD: metabolic dysfunction-associated steatotic liver disease; SLD: steatotic liver disease; HTN: hypertension; ORs: odds ratios; CI: confidence interval; CMRF: cardiometabolic risk factor; CMD: cardiometabolic dysfunction.  ^a^ Adjusted for age; sex; smoking; AST and ALT levels  ^b^ Minimal cardiometabolic dysfunction was defined as meeting 0 to 1 cardiometabolic criteria; Moderate cardiometabolic dysfunction was defined as meeting 2 to 3 cardiometabolic criteria; Significant cardiometabolic dysfunction was defined as meeting 4 to 5 cardiometabolic criteria | | | | | | |

| **Supplementary Table 2. Factors associated with existing DM in the cross-sectional cohort** | | | | | | |
| --- | --- | --- | --- | --- | --- | --- |
|  | **Number** | | **Logistic regression** | | | |
|  | **Total, n** | **DM, n (%)** | **Univariate** | | **Multivariate^a^** | |
|  |  |  | **ORs (95% C.I.)** | ***p*-value** | **ORs (95% C.I.)** | ***p*-value** |
| **MASLD** |  |  |  |  |  |  |
| Non-SLD | 14694 | 188 (1.28) | 1 |  | 1 |  |
| MASLD | 16762 | 891 (5.32) | 4.33 (3.70–5.08) | **<0.0001** | 3.17 (2.69–3.73) | **<0.0001** |
| **Comparison among subgroups** | |  |  |  |  |  |
| Healthy control | 4835 | 0 (0.00) | - |  | - |  |
| Non-SLD/CMRF+ | 9859 | 188 (1.91) | 1 |  | 1 |  |
| Simple SLD | 1113 | 0 (0.00) | - |  | - |  |
| MASLD | 16762 | 891 (5.32) | 2.89 (2.46–3.39) | **<0.0001** | 2.39 (2.03–2.81) | **<0.0001** |
| **Grade of CMD in SLD^b^** |  |  |  |  |  |  |
| Minimal | 4564 | 20 (0.44) | 1 |  | 1 |  |
| Moderate | 10694 | 413 (3.86) | 9.13 (5.82–14.32) | **<0.0001** | 7.27 (4.62–11.44) | **<0.0001** |
| Significant | 2617 | 458 (17.50) | 48.20 (30.71–75.64) | **<0.0001** | 32.20 (20.42–50.79) | **<0.0001** |
| Abbreviations: MASLD: metabolic dysfunction-associated steatotic liver disease; SLD: steatotic liver disease; DM: diabetes; ORs: odds ratios; CI: confidence interval; CMRF: cardiometabolic risk factor; CMD: cardiometabolic dysfunction.  ^a^ Adjusted for age; sex; smoking; and AST and ALT levels  ^b^ Minimal cardiometabolic dysfunction was defined as meeting 0 to 1 cardiometabolic criteria; Moderate cardiometabolic dysfunction was defined as meeting 2 to 3 cardiometabolic criteria; Significant cardiometabolic dysfunction was defined as meeting 4 to 5 cardiometabolic criteria | | | | | | |

| **Supplementary Table 3. Correlation between the number of cardiometabolic components of MASLD and existing HTN and DM in the cross-sectional cohort** | | | | | | |
| --- | --- | --- | --- | --- | --- | --- |
| **Number of CMRF** | **Number** | | **Logistic regression** | | | |
|  | **Total, n** | **HTN / DM,**  **n (%)** | **Univariate** | | **Multivariate^a^** | |
|  |  |  | **ORs (95% CI)** | ***p*-value** | **ORs (95% CI)** | ***p*-value** |
| **HTN** | | | | | | |
| 0 | 1113 | 0 (0.00) | -- |  | -- |  |
| 1 | 3451 | 69 (2.00) | 1 |  | 1 |  |
| 2 | 5595 | 809 (14.46) | 8.29 (6.45–10.64) | **<0.0001** | 7.48 (5.82–9.61) | **<0.0001** |
| 3 | 5099 | 1410 (27.65) | 18.73 (14.65–23.96) | **<0.0001** | 15.62 (12.19–20.01) | **<0.0001** |
| 4 | 2374 | 956 (40.27) | 33.05 (25.68–42.52) | **<0.0001** | 25.70 (19.93–33.15) | **<0.0001** |
| 5 | 243 | 108 (44.44) | 39.21 (27.70–55.51) | **<0.0001** | 28.67 (20.14–40.82) | **<0.0001** |
| Total | 17875 | 3352 (18.75) |  |  |  |  |
| **DM** |  |  |  |  |  |  |
| 0 | 1113 | 0 (0.00) | -- |  | -- |  |
| 1 | 3451 | 20 (0.58) | 1 |  | 1 |  |
| 2 | 5595 | 93 (1.66) | 2.90 (1.79–4.71) | **<0.0001** | 2.54 (1.56–4.14) | **0.0002** |
| 3 | 5099 | 320 (6.28) | 11.49 (7.30–18.08) | **<0.0001** | 8.99 (5.70–14.20) | **<0.0001** |
| 4 | 2374 | 405 (17.06) | 35.29 (22.45–55.47) | **<0.0001** | 25.05 (15.87–39.55) | **<0.0001** |
| 5 | 243 | 53 (21.81) | 47.85 (28.04–81.68) | **<0.0001** | 30.13 (17.52–51.84) | **<0.0001** |
| Total | 17875 | 891 (4.98) |  |  |  |  |
| Abbreviations: MASLD: metabolic dysfunction-associated steatotic liver disease; HTN: hypertension; DM: diabetes; ORs: odds ratios; CI: confidence interval; CMRF: cardiometabolic risk factor  ^a^ Adjusted for age; sex; smoking; and AST and ALT levels | | | | | | |

| **Supplementary Table 4. Cumulative lifetime risk and incidence of new-onset HTN and DM in the longitudinal cohort** | | | | | | | | | | |
| --- | --- | --- | --- | --- | --- | --- | --- | --- | --- | --- |
|  | **Annual incidence rate (per 1000 person-years)** | **Lifetime Risk (%)  Age (years old)** | | | | **Cumulative Incidence (%)  Follow-up (years)** | | | | |
|  |  | **40** | **50** | **60** | **65** | **3 y** | **5 y** | **7 y** | **10 y** | **14 y** |
| Hypertension (n=2,358) | 13.8 | 1.31 | 8.31 | 23.49 | 32.70 | 2.49 | 5.34 | 8.68 | 14.10 | 20.56 |
| Diabetes  (n=601) | 3.4 | 0.26 | 1.79 | 6.70 | 10.90 | 0.59 | 1.34 | 2.26 | 3.82 | 5.19 |
| Abbreviations: HTN: hypertension, DM: diabetes, No.: number, y: years | | | | | | | | | | |

| **Supplementary Table 5. Cumulative incidence of new-onset HTN** | | | | | | | |
| --- | --- | --- | --- | --- | --- | --- | --- |
| **Comparison among subgroups** | **Cumulative Incidence (%)  Follow-up (years)** | | | | | **Crude HR (95% CI)  *p-*value** | **Adjusted HR^a^ (95% CI)  *p-*value** |
|  | **3 y** | **5 y** | **7 y** | **10 y** | **14 y** |  |  |
| Healthy control | 0.18 | 0.34 | 0.68 | 1.08 | 3.66 | 1 | 1 |
| Non-SLD/CMRF+ | 2.21 | 4.88 | 8.03 | 13.07 | 19.07 | 8.85 (6.41–12.22)  **<0.0001** | 7.48 (5.41–10.34)  **<0.0001** |
| Simple SLD | 0.49 | 0.66 | 1.64 | 2.50 | 4.09 | 1.69 (0.94–3.02)  0.0782 | 1.66 (0.93–2.97)  0.0878 |
| MASLD | 3.68 | 7.72 | 12.25 | 17.32 | 27.42 | 13.59 (9.89–18.68)  **<0.0001** | 10.71 (7.78–14.74)  **<0.0001** |
| Abbreviations: SLD: steatotic liver disease; HTN: hypertension; HR: hazard ratio; CI: confidence interval; y: years; CMRF: cardiometabolic risk factor  ^a^ Adjusted for age; sex; smoking; and AST and ALT levels | | | | | | | |

| **Supplementary Table 6. Cumulative lifetime risk of new-onset HTN** | | | | | | |
| --- | --- | --- | --- | --- | --- | --- |
| **Comparison among subgroups** | **Lifetime Risk (%)  Age (years old)** | | | | **Crude HR (95% CI)  *p*-value** | **Adjusted HR^a^ (95% CI)  *p*-value** |
|  | **40** | **50** | **60** | **65** |  |  |
| Healthy control | 0.16 | 0.95 | 3.92 | 5.60 | 1 | 1 |
| Non-SLD/CMRF+ | 1.10 | 7.52 | 22.27 | 32.23 | 7.05 (5.11–9.74)  **<0.0001** | 6.39 (4.62–8.82)  **<0.0001** |
| Simple SLD | 0.13 | 1.39 | 5.86 | 15.27 | 1.60 (0.89–2.86)  0.1130 | 1.64 (0.91–2.93)  0.0972 |
| MASLD | 1.87 | 11.06 | 29.09 | 38.79 | 10.01 (7.28–13.75)  **<0.0001** | 8.66 (6.30–11.91)  **<0.0001** |
| Abbreviations: SLD: steatotic liver disease; HTN: hypertension; HR: hazard ratio; CI: confidence interval; CMRF: cardiometabolic risk factor  ^a^ Adjusted for sex; smoking; and AST and ALT levels | | | | | | |

| **Supplementary Table 7. Age-, sex-, and BMI-matched cohorts of MASLD vs non-SLD** | | | | | | |
| --- | --- | --- | --- | --- | --- | --- |
| **Variable** | **Original sample** | | | **Matched sample** | | |
|  | **Non-SLD (n=13,376)** | **MASLD**  **(n=12,620)** | ***p*-value** | **Non-SLD**  **(n=6,622)** | **MASLD (n=6,622)** | ***p*-value** |
| **Age** |  |  | **<0.0001** |  |  | 0.8185 |
| ≤35 | 5605 (41.90) | 3779 (29.94) |  | 2237 (33.78) | 2237 (33.78) |  |
| 36-45 | 4807 (35.94) | 5250 (41.60) |  | 2602 (39.29) | 2589 (39.10) |  |
| 46-55 | 2557 (19.12) | 3126 (24.77) |  | 1536 (23.20) | 1566 (23.65) |  |
| 56-65 | 407 (3.04) | 465 (3.68) |  | 247 (3.73) | 230 (3.47) |  |
| **Sex** |  |  | **<0.0001** |  |  | 0.6569 |
| Female | 3931 (13.84) | 1747 (29.39) |  | 1106 (16.70) | 1087 (16.41) |  |
| Male | 9445 (86.16) | 10873 (70.61) |  | 5516 (83.30) | 5535 (83.59) |  |
| **BMI** |  |  | **<0.0001** |  |  | 1.0000 |
| <18.5 kg/m^2^ | 941 (7.03) | 29 (0.23) |  | 29 (0.44) | 29 (0.44) |  |
| 18.5-22.9 kg/m^2^ | 7302 (54.59) | 1608 (12.74) |  | 1608 (24.28) | 1608 (24.28) |  |
| 23-24.9 kg/m^2^ | 3179 (23.77) | 3839 (30.42) |  | 3031 (45.77) | 3031 (45.77) |  |
| >25 kg/m^2^ | 1954 (14.61) | 7144 (56.61) |  | 1954 (29.51) | 1954 (29.51) |  |
| **New-onset of HTN** | | | | | | |
| Number (Percentage) | 743 (5.55) | 1599 (12.67) |  | 523 (7.90) | 709 (10.71) |  |
| Annual Incidence  (Per-1000-person years) | 9.0 | 19.7 |  | 12.1 | 16.8 |  |
| Univariate HRs  (95% C.I.) | 1 | 2.17  (1.99–2.37) |  | 1 | 1.40  (1.25–1.57) |  |
| *p*-value |  | **<0.0001** |  |  | **<0.0001** |  |
| Multivariate HRs  (95% C.I.) | 1 | 1.89  (1.73–2.07)^a^ |  | **1** | 1.39  (1.24–1.56)^b^ |  |
| *p*-value |  | **<0.0001** |  |  | **<0.0001** |  |
| **New-onset of DM** | | | | | | |
| Number (Percentage) | 54 (0.40) | 545 (4.32) |  | 38 (0.57) | 206 (3.11) |  |
| Annual Incidence  (Per-1000-person years) | 0.6 | 6.3 |  | 0.8 | 4.6 |  |
| Univariate HRs  (95% C.I.) | 1 | 9.87  (7.47–13.06) |  | 1 | 5.54  (3.92–7.83) |  |
| *p*-value |  | **<0.0001** |  |  | **<0.0001** |  |
| Multivariate HRs  (95% C.I.) | 1 | 4.26  (3.15–5.76)^a^ |  | 1 | 5.28  (3.73–7.47)^b^ |  |
| *p*-value |  | **<0.0001** |  |  | **<0.0001** |  |
| Abbreviations: MASLD: metabolic dysfunction-associated steatotic liver disease; SLD: steatotic liver disease; HTN: hypertension; DM: diabetes; HRs: hazard ratios; CI: confidence interval; CMRF: cardiometabolic risk factor  ^a^ Adjusted for age; sex; smoking; and AST and ALT levels  ^b^ Adjusted for smoking; and AST and ALT levels | | | | | | |

| **Supplementary Table 8. Age-, sex-, and CMRF-matched cohorts of MASLD vs Non-SLD/CMRF+** | | | | | | |
| --- | --- | --- | --- | --- | --- | --- |
| **Variable** | **Original sample** | | | **Matched sample** | | |
|  | **Non-SLD/CMRF+ (n=8.547)** | **MASLD**  **(n=12,620)** | ***p*-value** | **Non-SLD/CMRF+**  **(n=7,214)** | **MASLD (n=7,214)** | ***p*-value** |
| **Age** |  |  | **<0.0001** |  |  | 0.6340 |
| ≤35 | 3160 (36.97) | 3779 (29.94) |  | 2525 (35.00) | 2454 (34.02) |  |
| 36-45 | 3200 (37.44) | 5250 (41.60) |  | 2779 (38.52) | 2826 (39.17) |  |
| 46-55 | 1874 (21.93) | 3126 (24.77) |  | 1642 (22.76) | 1654 (22.93) |  |
| 56-65 | 313 (3.66) | 465 (3.68) |  | 268 (3.71) | 280 (3.88) |  |
| **Sex** |  |  | **<0.0001** |  |  | 0.4028 |
| Female | 1652 (19.33) | 1747 (13.84) |  | 1266 (17.55) | 1228 (17.02) |  |
| Male | 6895 (80.67) | 10873 (86.18) |  | 5948 (82.45) | 5986 (82.98) |  |
| **CMRF** |  |  | **<0.0001** |  |  | 1.0000 |
| 1 | 4679 (54.74) | 3346 (26.51) |  | 3346 (46.38) | 3346 (46.38) |  |
| 2 | 2813 (32.91) | 4631 (36.70) |  | 2813 (38.99) | 2813 (38.99) |  |
| 3 | 885 (10.35) | 3370 (26.70) |  | 885 (12.27) | 885 (12.27) |  |
| 4 | 164 (1.92) | 1171 (9.28) |  | 164 (2.27) | 164 (2.27) |  |
| 5 | 6 (0.07) | 102 (0.81) |  | 6 (0.08) | 6 (0.08) |  |
| **New-onset of HTN** | | | | | | |
| Number (Percentage) | 704 (8.24) | 1599 (12.67) |  | 651 (9.02) | 621 (8.61) |  |
| Annual Incidence  (Per-1000-person years) | 12.9 | 19.7 |  | 13.9 | 13.5 |  |
| Univariate HRs  (95% C.I.) | 1 | 1.54  (1.41–1.68) |  | 1 | 0.97  (0.87–1.09) |  |
| *p*-value |  | **<0.0001** |  |  | 0.6390 |  |
| **New-onset of DM** | | | | | | |
| Number (Percentage) | 52 (0.61) | 545 (4.32) |  | 47 (0.65) | 155 (2.15) |  |
| Annual Incidence  (Per-1000-person years) | 0.9 | 6.3 |  | 1.0 | 3.2 |  |
| Univariate HRs  (95% C.I.) | 1 | 7.01  (5.28–9.32) |  | 1 | 3.42  (2.46–4.73) |  |
| *p*-value |  | **<0.0001** |  |  | **<0.0001** |  |
| Abbreviations: MASLD: metabolic dysfunction-associated steatotic liver disease; SLD: steatotic liver disease; HTN: hypertension; DM: diabetes; HRs: hazard ratios; CI: confidence interval; CMRF: cardiometabolic risk factor | | | | | | |

| **Supplementary Table 9. Correlation between the number of CMRF and incidence of new-onset HTN and DM in the longitudinal cohort** | | | | | | | | | | |
| --- | --- | --- | --- | --- | --- | --- | --- | --- | --- | --- |
| **Number of CMRF** | **New-onset HTN / DM** | | | **Cumulative Incidence (%)** | | | | | **Cox proportional regression** | |
|  | **Total, n** | **Yes, n (%)** | **Annual Incidence**  **(Per 1000 person-years)** | **Follow-up (years)** | | | | | **Univariate HRs**  **(95% CI)** | **Multivariate HRs^a^**  **(95% CI)** |
|  |  |  |  | **3 y** | **5 y** | **7 y** | **10 y** | **14 y** |  |  |
| **HTN** | | | |  |  |  |  |  |  |  |
| 0 | 1113 | 16 (1.44) | 2.4 | 0.49 | 0.66 | 1.64 | 2.50 | 4.09 | 1 | 1 |
| 1 | 3346 | 116 (3.47) | 5.4 | 0.82 | 1.65 | 2.87 | 5.41 | 9.43 | 2.22 (1.31–3.74)  **0.0028** | 2.02 (1.20–3.41)  **0.0086** |
| 2 | 4631 | 522 (11.27) | 17.1 | 2.90 | 6.47 | 10.56 | 17.24 | 24.50 | 6.95 (4.23–11.44)  **<0.0001** | 6.11 (3.71–10.08)  **<0.0001** |
| 3 | 3370 | 637 (18.90) | 29.4 | 5.68 | 11.59 | 17.90 | 27.50 | 38.31 | 12.04 (7.33–19.77)  **<0.0001** | 10.31 (6.26–16.99)  **<0.0001** |
| 4 | 1171 | 302 (25.79) | 42.6 | 8.41 | 16.85 | 25.44 | 39.28 | 51.04 | 17.92 (10.84–29.62)  **<0.0001** | 14.95 (9.01–24.81)  **<0.0001** |
| 5 | 102 | 22 (21.57) | 40.8 | 7.95 | 15.67 | 28.98 | 42.36 | -- | 18.23 (9.58–34.72)  **<0.0001** | 15.53 (8.13–29.65)  **<0.0001** |
| Total | 13733 | 1615 (11.76) |  |  |  |  |  |  |  |  |
| **DM** | | | |  |  |  |  |  |  |  |
| 0 | 1113 | 2 (0.18) | 0.3 | 0.00 | 0.00 | 0.22 | 0.22 | 1.75 | 1 | 1 |
| 1 | 3346 | 22 (0.66) | 1.0 | 0.21 | 0.35 | 0.66 | 0.93 | 1.88 | 3.32 (0.78–14.14)  0.1038 | 2.92 (0.68–12.42)  0.1477 |
| 2 | 4631 | 92 (1.99) | 2.8 | 0.70 | 1.41 | 1.79 | 3.19 | 4.01 | 9.16 (2.26–37.19)  **0.0019** | 7.64 (1.88–31.08)  **<0.0001** |
| 3 | 3370 | 206 (6.11) | 8.7 | 1.54 | 3.19 | 5.67 | 9.21 | 12.55 | 27.86 (6.92–112.14)  **<0.0001** | 21.89 (5.42–88.41)  **<0.0001** |
| 4 | 1171 | 208 (17.76) | 27.5 | 3.87 | 9.70 | 16.58 | 28.65 | 37.45 | 90.89 (22.58–363.83)  **<0.0001** | 67.87 (16.80–274.27)  **<0.0001** |
| 5 | 102 | 17 (16.67) | 30.6 | 4.38 | 9.10 | 16.09 | 43.10 | -- | 108.81 (25.13–471.03)  **<0.0001** | 82.38 (18.98–357.58)  **<0.0001** |
| Total | 13733 | 547 (3.98) |  |  |  |  |  |  |  |  |
| Abbreviations: MASLD: metabolic dysfunction-associated steatotic liver disease; HTN: hypertension; DM: diabetes; HRs: hazard ratios; CI: confidence interval; CMRF: cardiometabolic risk factor  ^a^ Adjusted for age; sex; smoking; and AST and ALT levels | | | | | | | | | | |

| **Supplementary Table 10. New-onset HTN in longitudinal cohorts across cardiometabolic risk transition groups** | | | | | |
| --- | --- | --- | --- | --- | --- |
| **Changes of grade in CMD during follow-up^a^** | **New-onset HTN** | | | **Cox proportional regression** | |
|  | **Total, n** | **Yes, n (%)** | **Annual Incidence  (Per 1000 person-years)** | **Crude HRs**  **(95% CI)** | ***p* value** |
| **Minimal cardiometabolic dysfunction** | | | | | |
| Minimal-Minimal | 2574 | 28 (1.09) | 1.8 | 1 |  |
| Minimal-Moderate | 1759 | 95 (5.40) | 8.0 | 4.18 (2.74–6.37) | **<0.0001** |
| Minimal-Significant | 126 | 9 (7.14) | 9.7 | 5.02 (2.37–10.63) | **<0.0001** |
| **Moderate cardiometabolic dysfunction** | | | | | |
| Moderate-Minimal | 1471 | 20 (1.36) | 2.0 | 0.07 (0.05–0.11) | **<0.0001** |
| Moderate-Moderate | 5613 | 960 (17.10) | 26.6 | 1 |  |
| Moderate-Significant | 917 | 179 (19.52) | 29.7 | 1.11 (0.95–1.30) | 0.1909 |
| **Significant cardiometabolic dysfunction** | | | | | |
| Significant-Minimal | 67 | 3 (4.48) | 7.9 | 0.13 (0.04–0.41) | **<0.0001** |
| Significant-Moderate | 665 | 132 (19.85) | 31.8 | 0.50 (0.40–0.63) | **<0.0001** |
| Significant-Significant | 541 | 189 (34.94) | 60.8 | 1 |  |
| Abbreviations: CMD: cardiometabolic dysfunction; HRs: hazard ratios; CI: confidence interval; HTN: hypertension  ^a^ “grade in CMD during follow-up” was defined as the grade if CMD at the time of onset of new HTN among participants. In cases where participants did not develop new-onset HTN, the count of cardiometabolic components recorded during their last visit was utilized.  ^b^ Minimal CMD was defined as meeting 0 or 1 cardiometabolic criteria; Moderate CMD was defined as meeting 2 or 3 cardiometabolic criteria; Significant CMD was defined as meeting 4 or 5 cardiometabolic criteria | | | | | |

| **Supplementary Table 11. Cumulative incidence of new-onset DM** | | | | | | | |
| --- | --- | --- | --- | --- | --- | --- | --- |
| **Comparison among subgroups** | **Cumulative Incidence (%)  Follow-up (years)** | | | | | **Crude HR (95% CI)  *p-*value** | **Adjusted HR^a^ (95% CI)  *p-*value** |
|  | **3 y** | **5 y** | **7 y** | **10 y** | **14 y** |  |  |
| Healthy control | 0.00 | 0.08 | 0.08 | 0.08 | 0.08 | 1 | 1 |
| Non-SLD/CMRF+ | 0.15 | 0.41 | 0.70 | 0.92 | 1.38 | 12.00 (2.92–49.27)  **0.0006** | 9.66 (2.35–39.71)  **0.0017** |
| Simple SLD | 0.00 | 0.00 | 0.22 | 0.22 | 1.75 | 4.07 (0.57–28.91)  0.1602 | 3.93 (0.55–27.87)  0.1715 |
| MASLD | 1.13 | 2.50 | 4.13 | 7.08 | 9.34 | 84.55 (21.09–338.95)  **<0.0001** | 63.07 (15.71–253.24)  **<0.0001** |
| Abbreviations: SLD: steatotic liver disease; DM: diabetes; HR: hazard ratio; CI: confidence interval; y: years; CMRF: cardiometabolic risk factor  ^a^ Adjusted for age; sex; smoking; and AST and ALT levels | | | | | | | |

| **Supplementary Table 12. Cumulative lifetime risk of new-onset DM** | | | | | | |
| --- | --- | --- | --- | --- | --- | --- |
| **Comparison among subgroups** | **Lifetime Risk (%)  Age (years old)** | | | | **Crude HR (95% CI)  *p-*v*a*lue** | **Adjusted HR^a^ (95% CI)  *p-*v*a*lue** |
|  | **40** | **50** | **60** | **65** |  |  |
| Healthy control | 0.00 | 0.10 | 0.10 | 0.10 | 1 | 1 |
| Non-SLD/CMRF+ | 0.07 | 0.46 | 1.79 | 2.93 | 9.35 (2.28–38.39)  **0.0019** | 8.47 (2.06–34.81)  **0.0030** |
| Simple SLD | 0.00 | 0.15 | 0.15 | 11.25 | 3.93 (0.55–27.91)  0.1710 | 3.99 (0.56–28.35)  0.1662 |
| MASLD | 0.47 | 3.13 | 11.31 | 17.69 | 62.11 (15.49–248.98)  **<0.0001** | 52.17 (13.00–209.32)  **<0.0001** |
| Abbreviations: SLD: steatotic liver disease; DM: diabetes; HR: hazard ratio; CI: confidence interval; CMRF: cardiometabolic risk factor  ^a^ Adjusted for sex; smoking; and AST and ALT levels | | | | | | |

| **Supplementary Table 13. New-onset DM in longitudinal cohorts across cardiometabolic risk transition groups** | | | | | |
| --- | --- | --- | --- | --- | --- |
| **Changes of grade in CMD during follow-up^a^** | **New-onset DM** | | | **Cox proportional regression** | |
|  | **Total, n** | **Yes, n (%)** | **Annual Incidence  (Per 1000 person-years)** | **Crude HRs**  **(95% CI)** | ***p* value** |
| **Minimal cardiometabolic dysfunction** | | | | | |
| Minimal-Minimal | 2564 | 5 (0.20) | 0.3 | 1 |  |
| Minimal-Moderate | 1761 | 15 (0.85) | 1.2 | 3.61 (1.31–9.94) | **0.0131** |
| Minimal-Significant | 134 | 4 (2.99) | 3.8 | 11.27 (3.02–42.03) | **0.0003** |
| **Moderate cardiometabolic dysfunction** | | | | | |
| Moderate-Minimal | 1436 | 7 (0.49) | 0.7 | 0.14 (0.06–0.29) | **<0.0001** |
| Moderate-Moderate | 5582 | 205 (3.67) | 5.2 | 1 |  |
| Moderate-Significant | 983 | 86 (8.75) | 11.9 | 2.25 (1.75–2.90) | **<0.0001** |
| **Significant cardiometabolic dysfunction** | | | | | |
| Significant-Minimal | 65 | 1 (0.17) | 2.5 | 0.06 (0.01–0.42) | **0.0048** |
| Significant-Moderate | 641 | 65 (10.14) | 15.9 | 0.36 (0.27–0.48) | **<0.0001** |
| Significant-Significant | 567 | 159 (28.04) | 43.6 | 1 |  |
| Abbreviations: CMD: cardiometabolic dysfunction; HRs: hazard ratios; CI: confidence interval; DM: diabetes  ^a^ “grade in CMD during follow-up” was defined as the grade of CMD at the time of onset of new DM among participants. In cases where participants did not develop new-onset DM, the count of cardiometabolic components recorded during their last visit was utilized.  ^b^ Minimal CMD was defined as meeting 0 or 1 cardiometabolic criteria; Moderate CMD was defined as meeting 2 or 3 cardiometabolic criteria; Significant CMD was defined as meeting 4 or 5 cardiometabolic criteria | | | | | |

| **Supplementary Table 14. Cumulative incidence of new-onset HTN and DM across cardiometabolic risk groups** | | | | | | | |
| --- | --- | --- | --- | --- | --- | --- | --- |
| **Cardiometabolic risk^a^** | **Cumulative Incidence (%)** | | | | | **Cox proportional regression** | |
|  | **Follow-up (years)** | | | | | **Univariate HRs**  **(95% CI)**  ***p-value*** | **Multivariate HRs^b^**  **(95% CI)**  ***p-value*** |
|  | **3 y** | **5 y** | **7 y** | **10 y** | **14 y** |  |  |
| **HTN** | | | | | |  |  |
| Minimal cardiometabolic dysfunction | 0.73 | 1.41 | 2.57 | 4.74 | 8.3 | 1 | 1 |
| Moderate cardiometabolic dysfunction | 4.08 | 8.65 | 13.71 | 21.66 | 30.39 | 9.61 (8.35–11.05)  **<0.0001** | 8.69 (7.55–10.00)  **<0.0001** |
| Significant cardiometabolic dysfunction | 8.37 | 16.75 | 25.65 | 39.47 | 50.81 | 16.94 (14.48–19.83)  **<0.0001** | 15.01 (12.81–17.59)  **<0.0001** |
| **DM** | | | | | |  |  |
| Minimal cardiometabolic dysfunction | 0.16 | 0.27 | 0.55 | 0.76 | 1.82 | 1 | 1 |
| Moderate cardiometabolic dysfunction | 1.06 | 2.17 | 3.46 | 5.81 | 7.69 | 9.29 (6.64–13.00)  **<0.0001** | 8.01 (5.71–11.22)  **<0.0001** |
| Significant cardiometabolic dysfunction | 3.91 | 9.66 | 16.52 | 29.21 | 38.14 | 45.86 (32.64–64.43)  **<0.0001** | 36.97 (26.25–52.06)  **<0.0001** |
| Abbreviations: HTN: hypertension; DM: diabetes; y: years; HRs: hazard ratios; CI: confidence interval  ^a^ Minimal cardiometabolic dysfunction was defined as meeting 0 to 1 cardiometabolic criteria; Moderate cardiometabolic dysfunction was defined as meeting 2 to 3 cardiometabolic criteria; Significant cardiometabolic dysfunction was defined as meeting 4 to 5 cardiometabolic criteria  ^b^ Adjusted for age; sex; smoking; and AST and ALT levels | | | | | | | |

| **Supplementary Table 15. Cumulative incidence of new-onset HTN and DM across cardiometabolic risk transition groups** | | | | | | | | | | |
| --- | --- | --- | --- | --- | --- | --- | --- | --- | --- | --- |
| **Changes of cardiometabolic risk during follow-up^ab^** | **Cumulative Incidence (%) of HTN Follow-up (years)** | | | | | **Cumulative Incidence (%) of DM Follow-up (years)** | | | | |
|  | **3 y** | **5 y** | **7 y** | **10 y** | **14 y** | **3 y** | **5 y** | **7 y** | **10 y** | **14 y** |
| **Minimal cardiometabolic dysfunction at baseline** | | | | | | | | | | |
| Minimal-Minimal | 0.35 | 0.63 | 1.53 | 1.89 | 2.88 | 0.08 | 0.08 | 0.17 | 0.17 | 0.63 |
| Minimal-Moderate | 0.98 | 2.22 | 3.67 | 7.89 | 14.00 | 0.19 | 0.45 | 0.90 | 1.08 | 2.81 |
| Minimal-Significant | 4.39 | 4.39 | 5.78 | 7.43 | 13.4 | 0.83 | 0.83 | 1.94 | 4.98 | 4.98 |
| **Moderate cardiometabolic dysfunction** | | | | | | | | | | |
| Moderate-Minimal | 0.25 | 1.10 | 1.53 | 2.27 | 2.90 | 0.09 | 0.50 | 0.50 | 0.69 | 1.05 |
| Moderate-Moderate | 4.90 | 10.01 | 16.00 | 25.35 | 35.73 | 1.05 | 2.19 | 3.56 | 5.63 | 7.42 |
| Moderate-Significant | 5.18 | 11.94 | 18.06 | 28.13 | 40.05 | 2.47 | 4.35 | 6.71 | 12.89 | 17.28 |
| **Significant cardiometabolic dysfunction** | | | | | | | | | | |
| Significant-Minimal | 3.08 | 3.08 | 3.08 | 9.54 | 9.54 | 0.00 | 0.00 | 0.00 | 0.00 | 9.09 |
| Significant-Moderate | 5.72 | 10.52 | 17.82 | 30.99 | 43.18 | 1.99 | 5.56 | 9.00 | 17.97 | 23.11 |
| Significant-Significant | 12.20 | 25.63 | 37.06 | 52.07 | 62.90 | 6.47 | 15.02 | 25.68 | 42.54 | 53.58 |
| Abbreviations: HTN: hypertension; DM: diabetes; y: years  ^a^ “Cardiometabolic risk during follow-up” was defined as the number of cardiometabolic components (range: 0-5) at the time of onset of new HTN and DM among participants. In cases where participants did not develop new-onset HTN and DM, the count of cardiometabolic components recorded during their last visit was utilized.  ^b^ Minimal cardiometabolic dysfunction was defined as meeting 0 to 1 cardiometabolic criteria; Moderate cardiometabolic dysfunction was defined as meeting 2 to 3 cardiometabolic criteria; Significant cardiometabolic dysfunction was defined as meeting 4 to 5 cardiometabolic criteria | | | | | | | | | | |
